# Supplementary material for: Molecular architecture of the DNA-binding sites of the P-loop ATPases MipZ and ParA from Caulobacter crescentus
Source: Nucleic Acids Res. 2020 Mar 30;48(9):4769–79. doi: 10.1093/nar/gkaa192 (PMC7229837; doi:10.1093/nar/gkaa192)
Supplement: gkaa192_Supplemental_Files [file gkaa192_supplemental_files.zip › Supplemental_information_.pdf]

SUPPLEMENTAL INFORMATION

Molecular architecture of the DNA-binding sites of the P-loop ATPases MipZ and ParA from *Caulobacter crescentus*

Laura Corrales-Guerrero, Binbin He, Yacine Refes, Gaël Panis, Gert Bange, Patrick Viollier,
Wieland Steinchen, and Martin Thanbichler

Address correspondence to
Wieland Steinchen, email: wieland.steinchen@synmikro.uni-marburg.de
Martin Thanbichler, email: thanbichler@uni-marburg.de

This PDF file includes:

Figures S1 to S15
Tables S1 to S3
Legend to Datasets S1 and S2
SI References

SUPPLEMENTAL FIGURES

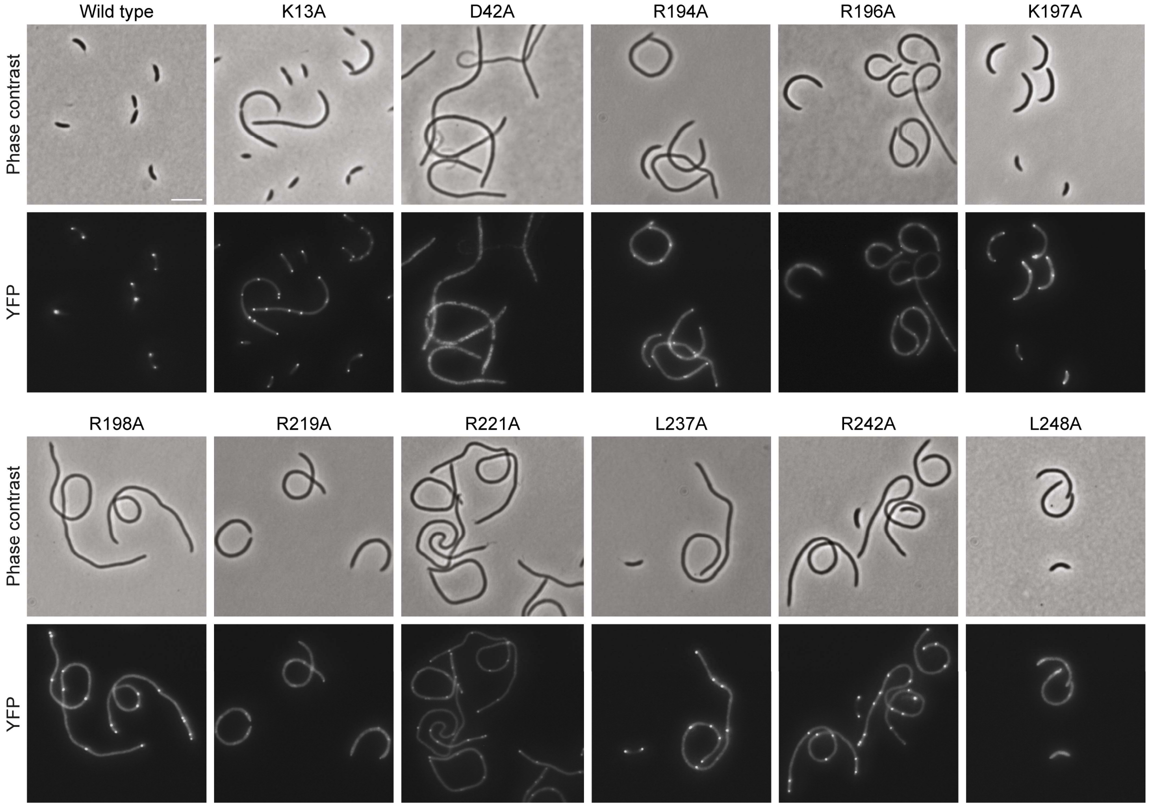

Figure S1. **Phenotype of *C. crescentus* strains producing MipZ-eYFP variants with reduced DNA-binding activity.** Strains BH64 (WT), BH100 (K13A), BH99 (D42A), BH84 (R194A), BH120 (R196A), BH85 (K197A), BH86 (R198A), BH89 (R219A), BH121 (R221A), BH91 (L237A), BH92 (R242A) and BH94 (L248A) were pre-grown in PYE medium containing 0.5 mM vanillate, washed, and then cultivated for 7 h in PYE medium containing 0.3 % xylose to deplete wild-type MipZ and induce the fluorescent protein fusions. Scale bar: 5 µm.

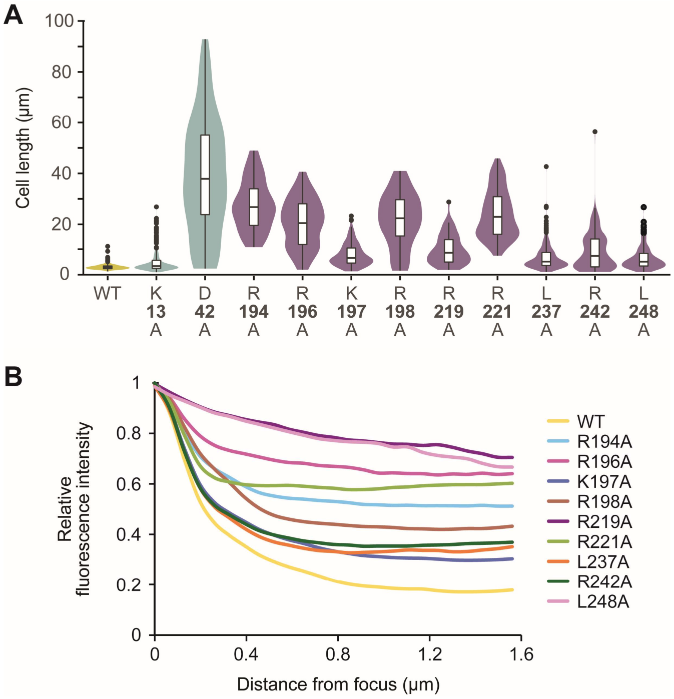

Figure S2. **Characterization of mutant strains producing MipZ-eYFP variants with impaired DNA-binding activity.** (**A**) Distribution of cell lengths in the cultures analyzed in **Fig. S1**. The data are represented as box plots, with the thick horizontal line indicating the median, the box the interquartile range, and the whiskers extending to the lowest and highest value within 1.5 times the interquartile range from the hinges, respectively. In addition, rotated kernel density plots are given for each dataset to indicate the distribution of the data. Number of cells analyzed: WT (343 cells), K13A (87 cells), D42A (268 cells), R194A (42 cells), R196A (56 cells), K197A (268 cells), R198A (84 cells), R219A (65 cells), R221A (96 cells), L237A (312 cells), R242A (199 cells) and L248A (234 cells). (**B**) Subcellular distribution of DNA-binding-defective MipZ-eYFP variants. Shown are normalized fluorescence intensity profiles giving the distribution of eYFP fluorescence along the long axis of the cell, starting at the brightest point of a polar (ParB-associated) focus and extending towards the cell center. Each curve represents the average of the profiles obtained from 108-140 cells (with the exception of R198A: 53 cells).

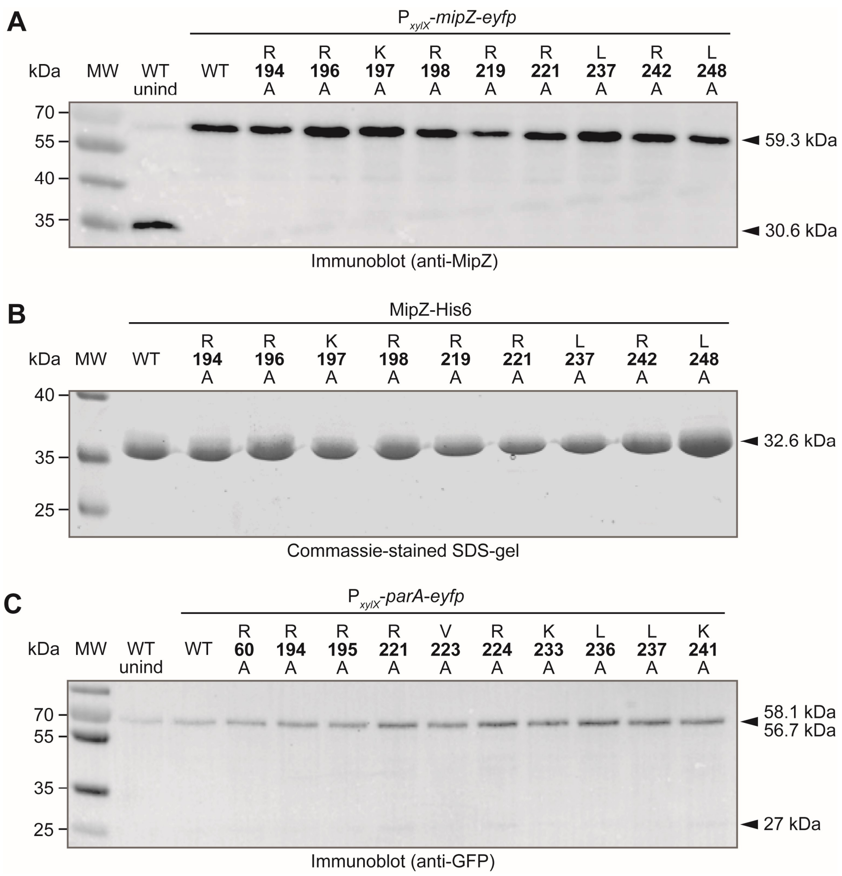

Figure S3. Stability and purity of the proteins analyzed in this study. (**A**) Immunoblot analysis of *C. crescentus* strains producing MipZ-eYFP or its DNA-binding-defective variants. Cells of strains BH64 (WT), BH84 (R194A), BH120 (R196A), BH85 (K197A), BH86 (R198A), BH89 (R219A) BH121 (R221A), BH91 (L237A), BH92 (R242A) and BH94 (L248A) were cultivated as described in **Fig. S1** and subjected to immunoblot analysis with an anti-MipZ antibody. Strain BH64 grown in vanillate-containing medium lacking xylose was analyzed as a control (unind). A mixture of standard proteins (MW) was applied as a reference. The molecular weights of the standard proteins are given on the left. The molecular weights of the fusion proteins and wild-type MipZ are indicated on the right. (**B**) Wild-type (WT) MipZ or the indicated mutant derivatives were purified and subjected to SDS-PAGE. Proteins were detected by staining with Coomassie Brilliant Blue R-250. (**C**) Immunoblot analysis of *C. crescentus* strains producing ParA-eYFP or different DNA-binding-defective variants. Cells of strains LC40 (WT), LC41 (R61A), LC42 (R194A), LC43 (R195A), LC44 (R221A), LC45 (V223A) LC46 (R224A), LC47 (K233A), LC48 (L236A), LC49 (L237A) and LC50 (K241A) were cultivated as described in **Fig. S8** and subjected to immunoblot analysis with an anti-GFP antibody. An uninduced culture was analyzed as a control. The molecular weights of the fusion proteins and free eYFP are indicated on the right.

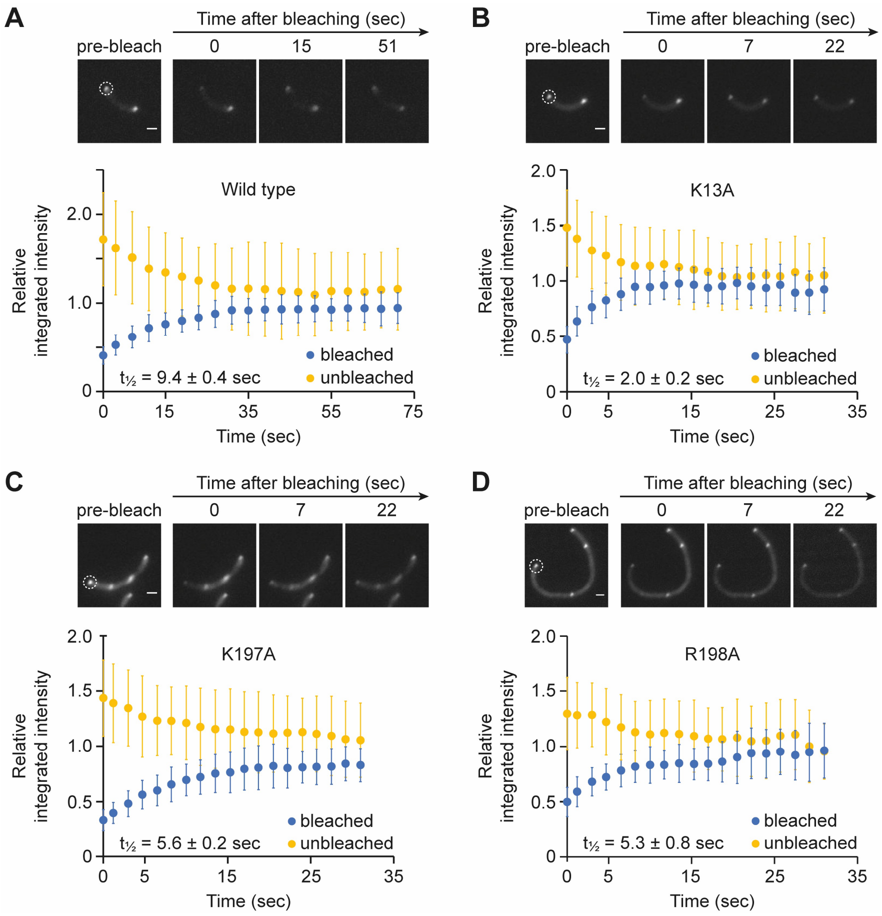

Figure S4. **FRAP analysis of the mobility of different MipZ-eYFP fusions.** Cells of strains (**A**) BH64 (MipZ-WT), (**B**) BH100 (MipZ-K13A), (**C**) BH85 (MipZ-K197A) and (**D**) BH86 (MipZ-K198A) were pre-grown in PYE medium containing 0.5 mM vanillate, shifted to medium without vanillate for 4 h to deplete wild-type MipZ and then incubated for 1.5 h with 0.3 % xylose to induce the fusion protein. After transfer of the cells to an agarose pad, one of the polar (ParB-associated) foci was bleached by a short laser pulse, and the recovery of the signal was followed over time. *Upper panels*: Fluorescence images of a representative cell taken before and at the indicated times after application of the laser pulse. The white dashed circle indicates the bleached region. *Lower panels*: quantification of the fluorescence signal in the bleached region and an unbleached region at the opposite cell pole. The first measurement was made immediately after the laser pulse (t = 0 sec). Error bars represent the standard deviation. The recovery half-times (± SD) are given in the graphs. Number of cells analyzed: WT (21 cells), K13A (27 cells), K197A (29 cells), R198A (20 cells). Scale bars: 1 µm.

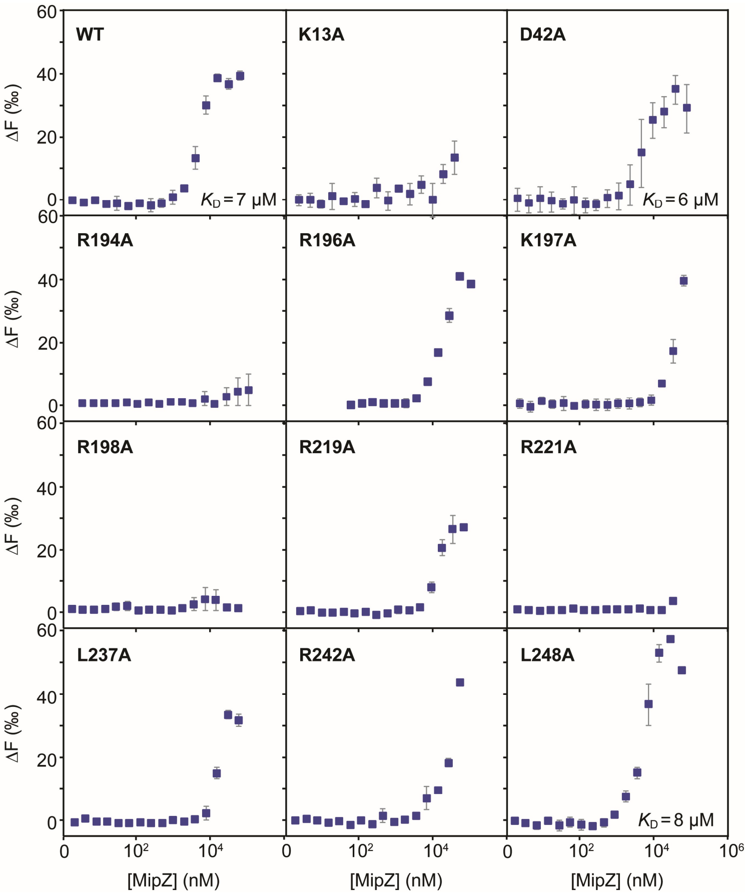

Figure S5. **Microscale thermophoresis analysis of the interaction of different MipZ variants with DNA.** A Cy3-labeled 26 bp dsDNA oligonucleotide (50 nM) was titrated with the indicated MipZ variants. For all binding curves reaching saturation, the corresponding equilibrium dissociation constant (K_D) is indicated in the graph.

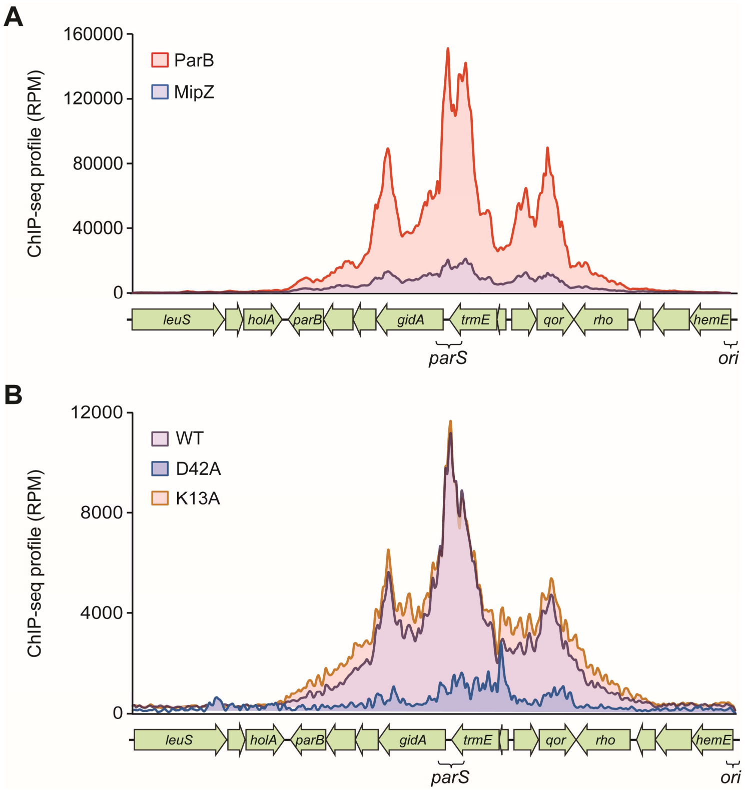

Figure S6. Association of MipZ with the chromosomal *parS* region. (A) ChIP-seq analysis of the interaction of wild-type MipZ and ParB with the *parS* region. Cells of wild-type strain NA1000 were fixed with formaldehyde and subjected to ChIP-seq analysis with anti-ParB or anti-MipZ antibodies. The graph shows the normalized number of reads mapping to the chromosomal region shown at the bottom. RPM: reads per million reads. (B) ChIP-seq analysis of different MipZ variants with the *parS* region. Cells of strains producing wild-type MipZ (BH64), MipZ-K13A (BH100) or MipZ-D42A (BH99) in place of the wild-type protein fixed with form-aldehyde and subjected to ChIP-seq analysis with anti-GFP antibodies. The data are presented as described in (A).

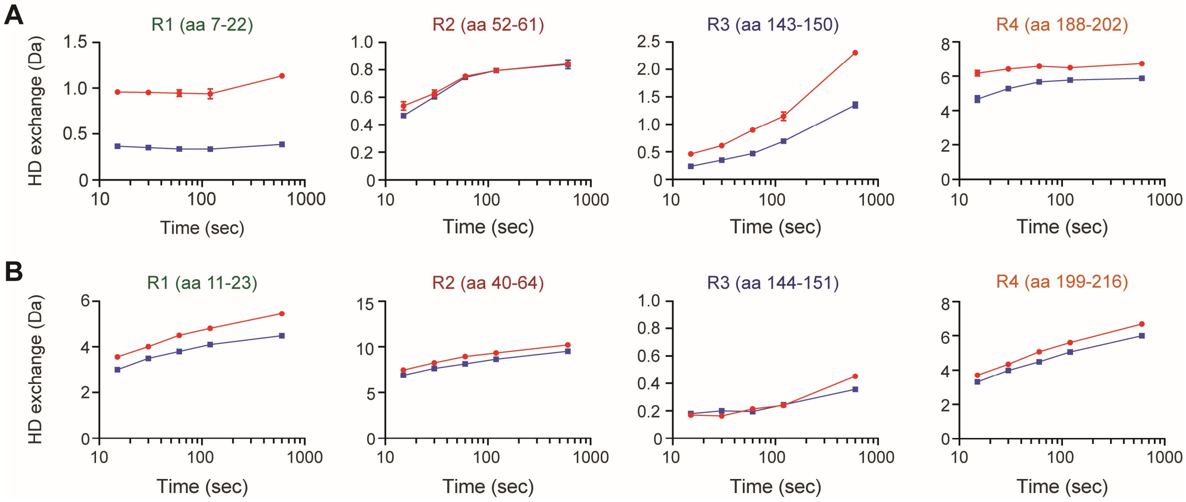

Figure S7. HDX analysis of the interaction of DNA with MipZ and ParA. The graphs show the average change in the mass of four representative peptides from regions R1, R2, R3 and R4 of (**A**) MipZ and (**B**) ParA (compare **Fig. 4 and 5**) after incubation of the proteins in deuterated buffer in the presence (blue) or absence (red) of a 14 bp dsDNA oligonucleotide (ran14-up/ran14-lo).

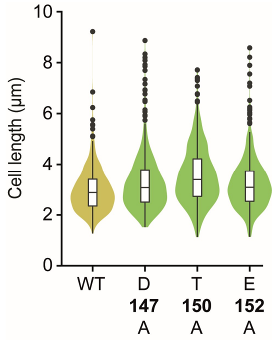

Figure S8. Characterization of strains producing MipZ variants with mutations in region R3. Strains BH78 (D147A), JAK10 (T150A) and BH79 (E152A) were pre-grown in PYE medium containing 0.5 mM vanillate, washed, and then cultivated for 7 h in PYE medium containing 0.3 % xylose to deplete wild-type MipZ and induce the fluorescent protein fusions. The graph shows the distribution of cell lengths in the three cultures (as defined in the legend to **Fig. S2A**). Number of cells analyzed: BH78 (585 cells), JAK10 (815 cells) and BH79 (639 cells).

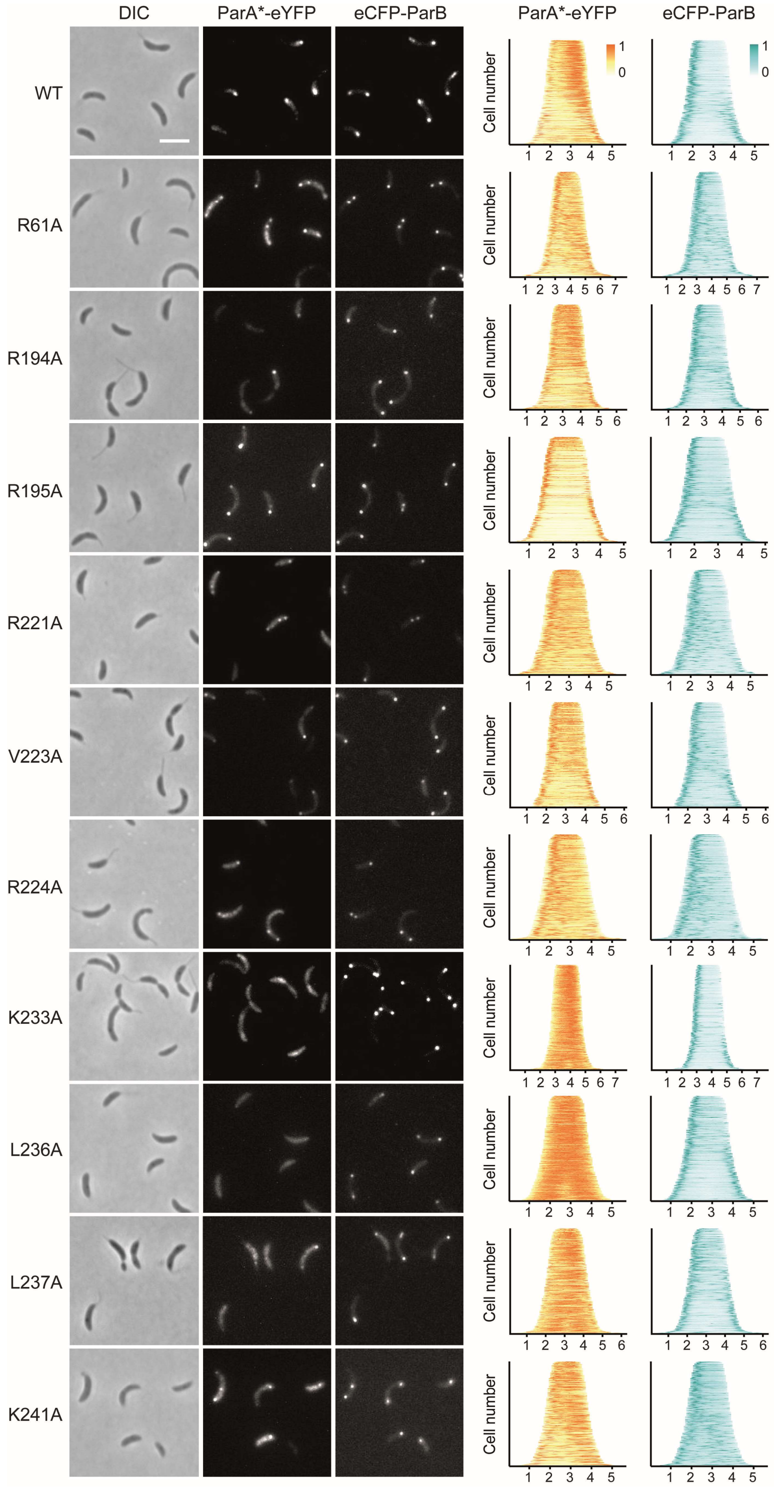

Figure S9. Localization pattern and function of ParA variants with mutations in the DNA-binding interface. Strains LC40 (WT), LC41 (R61A), LC42 (R194A), LC43 (R195A), LC44 (R221A), LC45 (V223A) LC46 (R224A), LC47 (K233A), LC48 (L236A), LC49 (L237A), and LC50 (K241A) were grown for 5 h in PYE medium containing 0.3 % xylose to induce the synthesis of the indicated ParA-eYFP variants. Subsequently, the cells were analyzed by phase contrast and fluorescence microscopy (scale bars: 3 µm). The demographs on the right show a quantification of the localization patterns of ParA-eYFP and eCFP-ParB in the different strains. To generate them, fluorescence intensity profiles obtained from a representative subpopulation of cells (n=350) were normalized, sorted according to cell length, and stacked on top of each other.

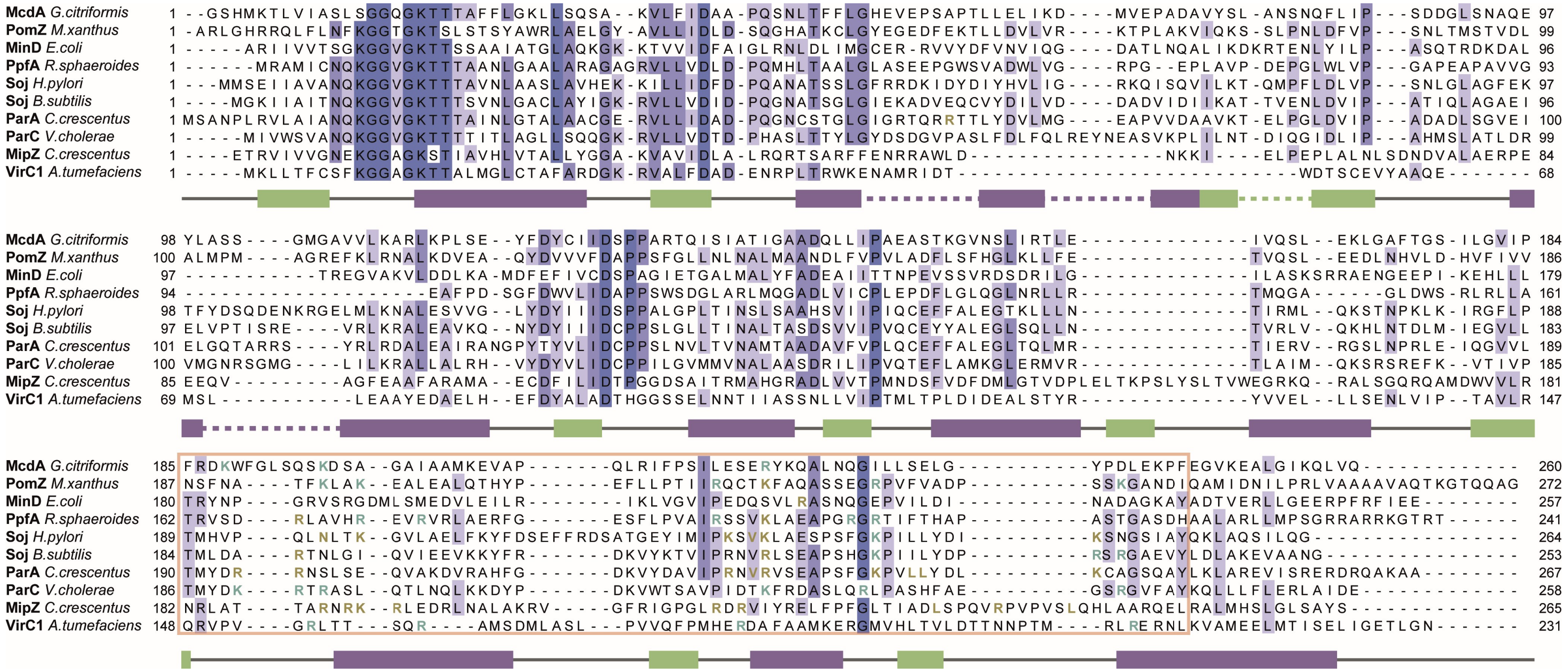

Figure S10. Amino acid sequence alignment of ParA-like ATPases. Shown is an alignment of the primary sequences of McdA (*G. citriformis*, UniProt accession number B7KMS4), PomZ (*M. xanthus*, Q1DEM0), MinD (*E. coli*, P0AEZ3), PpfA (*R. sphaeroides*, A3PKC4), ParA/Soj (*H. pylori*, O25759), ParA/Soj (*B. subtilis*, P37522), ParA (*C. crescentus*, B8GW31), ParC (*V. cholerae*, Q9KQD9), MipZ (*C. crescentus*, Q9A6C9) and VirC1 (*A. tumefaciens*, Q8VT89). The alignment was generated with PROMALS3D (1) and visualized with Jalview 2.8 (2). Conserved residues are highlighted in blue. Region R4 from Figures 4 and 5 is indicated by an orange box. Residues proven to be involved in DNA-binding are colored yellow. Residues predicted to have a role in DNA binding (see also Figure S12) are shown in cyan. Predicted secondary structural elements (obtained from PROMALS3D) are shown underneath the sequences in green (β-sheets) and purple (α-helices).

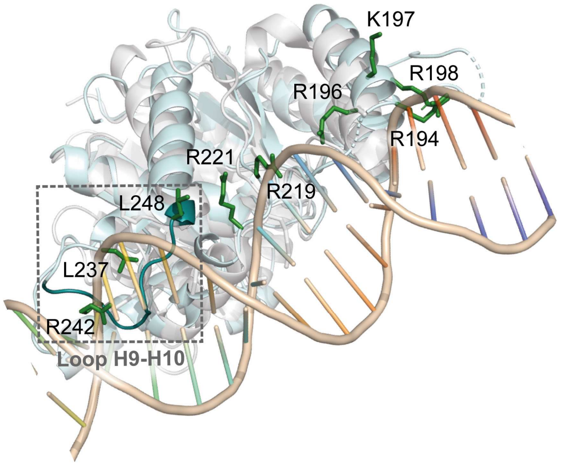

Figure S11. Superimposition of the crystal structures of MipZ and *Hp*ParA. Shown are single subunits from the crystal structures of the MipZ dimer (blue; PDB ID: 2XJ9) (3) and the *Hp*ParA•DNA complex (white/gold; PDB ID: 6IUC) (4). Amino acid residues mediating the DNA-binding activity of MipZ are colored blue. The loop between helices H9 and H10 of MipZ (dark green), containing residues L237, R242 and L248, is indicated by a dashed box.

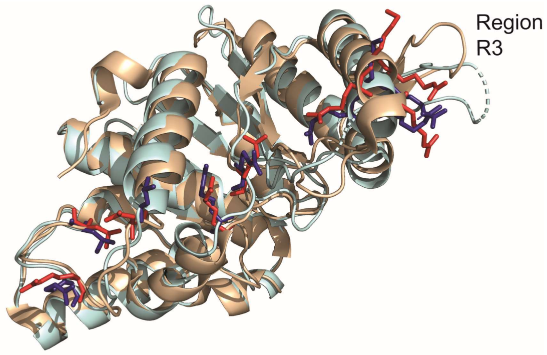

Figure S12. Comparison of the crystal structures of MipZ in the monomeric and dimeric state. Shown is a superimposition of monomeric MipZ (PDP ID: 2XJ4) (in wheat color, with DNA-binding residues colored red) and a single subunit of the MipZ dimer (PDB ID: 2XJ9) (in cyan, with DNA-binding residues colored blue) (3). The loop corresponding to region R3 of MipZ (see Figure 4) is indicated.

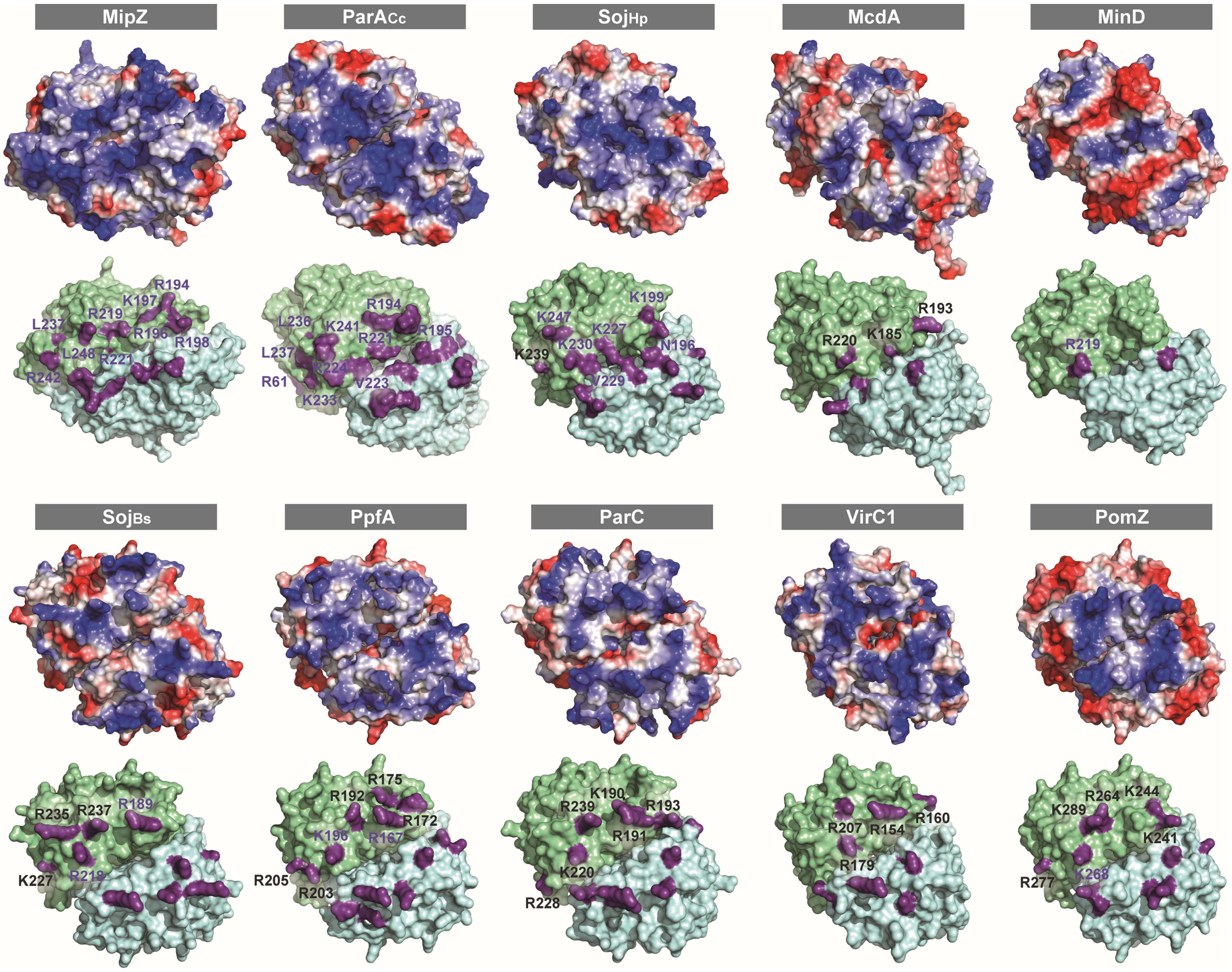

Figure S13. DNA-binding regions of various P-loop ATPases. Shown are the electrostatic surface potentials and surface structures of various ParA-like ATPases in the dimeric state. The analysis includes the crystal structures of MipZ (*C. crescentus*, PDB ID: 2XJ9) (3), ParA/Soj$_{Hp}$ (*H. pylori*, PDB ID: 6IUC) (4), McdA (*G. citriformis*, PDB ID: 6NOP) (5) and MinD (*E. coli*, PDB ID: 3Q9L) (6) as well as structural models of ParA$_{Cc}$ (*C. crescentus*), ParA/Soj$_{Bs}$ (*B. subtilis*), PpfA (*R. sphaeroides*), ParC (*V. cholerae*), VirC1 (*A. tumefaciens*) and PomZ (*M. xanthus*), generated with Phyre2 (7) using ParA/Soj$_{Hp}$ as a template. Positively charged residues located in regions corresponding to the DNA-binding interfaces of MipZ and *Cc*ParA are highlighted in purple. Residues that have been previously proven to be involved in DNA binding are labeled in purple, whereas newly predicted DNA-binding residues are labeled in black.

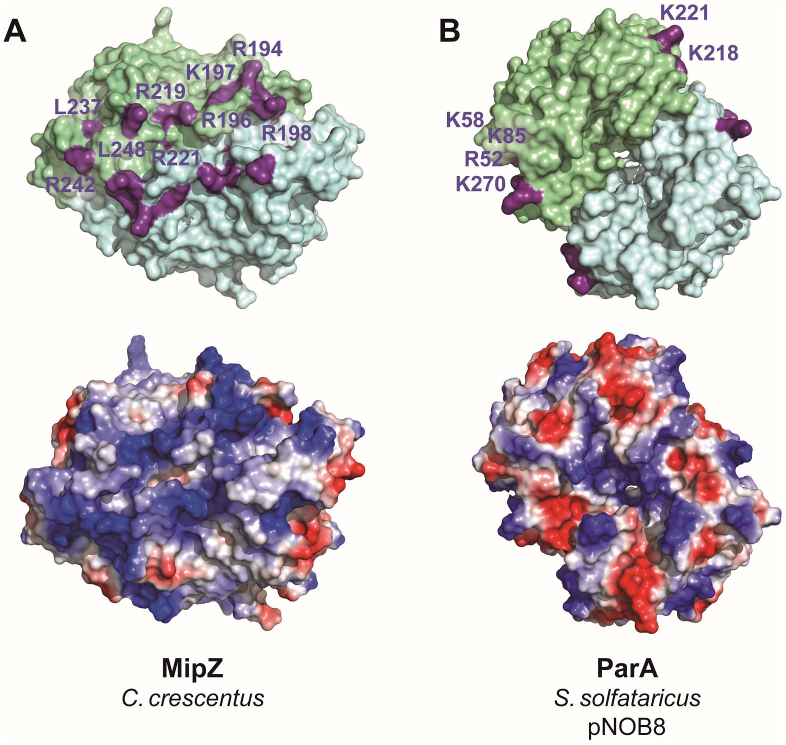

Figure S14. **DNA-binding interfaces of MipZ and pNOB8 ParA.** **(A,B)** Comparison of the crystal structures of dimeric complexes of (A) MipZ from *C. crescentus* (PDB ID: 2XJ9) (3) and (B) ParA from *S. solfataricus* pNOB8 (PDB ID: 5U1J) (8). The two subunits are shown in green and blue, respectively. Residues involved in DNA binding are highlighted in purple.

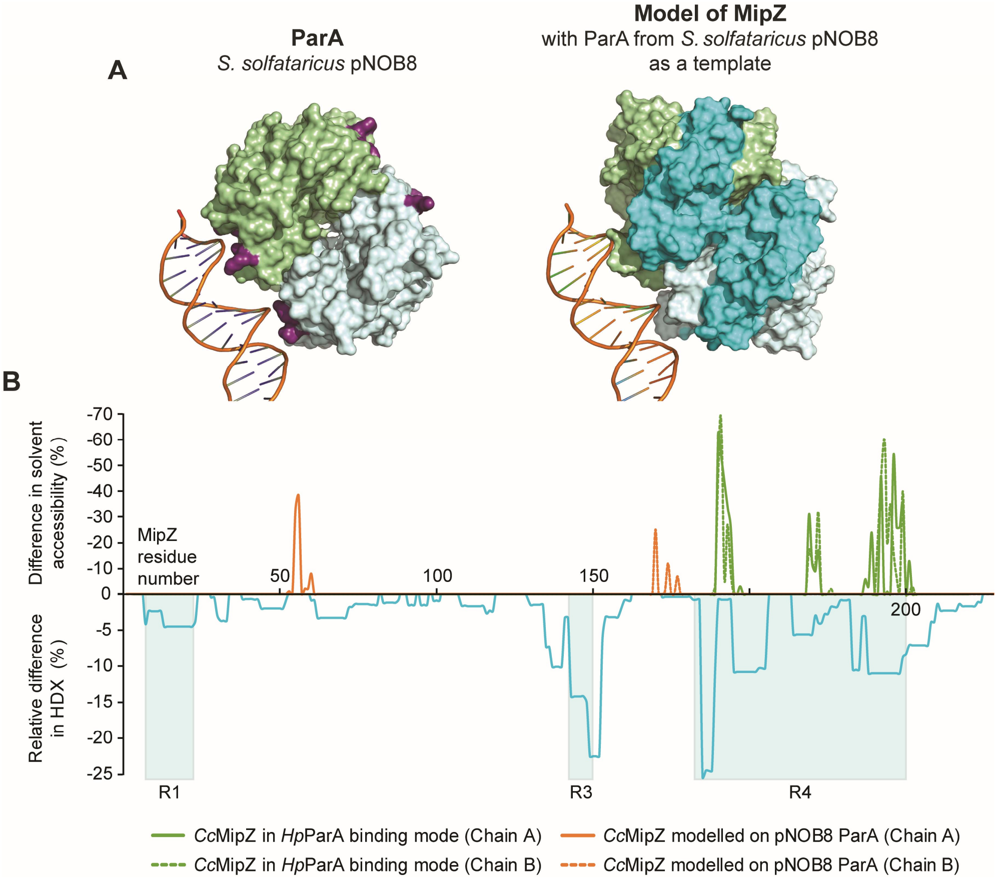

Figure S15. Assessment of the DNA-binding mode of MipZ. (A) Comparison of the crystal structure of the DNA-bound ParA dimer from *S. solfataricus* pNOB8 (PDB ID: 5U1J) with a model of the DNA-bound MipZ dimer generated with the structure of the pNOB8 ParA·DNA complex as a template. The two subunits of each protein are shown in green and blue, respectively. Regions of MipZ protected from hydrogen-deuterium exchange (HDX) upon addition of DNA (see Figure 4) are highlighted in cyan. (B) Comparison of the experimentally determined changes in HDX (cyan; see Figure 4) with the predicted changes in relative solvent accessibility induced by DNA binding to MipZ (PDB ID: 2XJ9) in *Hp*ParA binding mode (PDB ID: 6IUC) (green) and to a model of MipZ based on the structure of the pNOB8 ParA·DNA complex (PDB ID: 5U1J) (orange). Solvent accessibility was analyzed with PyMOL (Schrödinger, LLC). Note that in the pNOB8 ParA-based model, the DNA molecule is not in contact with the experimentally determined DNA-binding region. The structure of the pNOB8 ParA·DNA complex is thus not representative of the DNA complexes formed by MipZ and related P-loop ATPases.

SUPPLEMENTAL TABLES

Table S1. Strains used in this study.

Strain	Genotype/description	Source/Reference
C. crescentus		
CB15N	Wild-type strain (also known as NA1000)	(9)
MT190	*parB*::*ecfp-parB*	(10)
BH62	CB15N Δ*mipZ* P_{vanA}::P_{vanA}-*mipZ*	This study
BH64	CB15N Δ*mipZ* P_{vanA}::P_{vanA}-*mipZ* P_{xylX}::P_{xylX}-*mipZ-eyfp*	This study
BH78	CB15N Δ*mipZ* P_{vanA}::P_{vanA}-*mipZ* P_{xylX}::P_{xylX}-*mipZ*$_{D147A}$-*eyfp*	This study
BH79	CB15N Δ*mipZ* P_{vanA}::P_{vanA}-*mipZ* P_{xylX}::P_{xylX}-*mipZ*$_{E152A}$-*eyfp*	This study
BH84	CB15N Δ*mipZ* P_{vanA}::P_{vanA}-*mipZ* P_{xylX}::P_{xylX}-*mipZ*$_{R194A}$-*eyfp*	This study
BH85	CB15N Δ*mipZ* P_{vanA}::P_{vanA}-*mipZ* P_{xylX}::P_{xylX}-*mipZ*$_{K197A}$-*eyfp*	This study
BH86	CB15N Δ*mipZ* P_{vanA}::P_{vanA}-*mipZ* P_{xylX}::P_{xylX}-*mipZ*$_{R198A}$-*eyfp*	This study
BH89	CB15N Δ*mipZ* P_{vanA}::P_{vanA}-*mipZ* P_{xylX}::P_{xylX}-*mipZ*$_{R219A}$-*eyfp*	This study
BH91	CB15N Δ*mipZ* P_{vanA}::P_{vanA}-*mipZ* P_{xylX}::P_{xylX}-*mipZ*$_{L237A}$-*eyfp*	This study
BH92	CB15N Δ*mipZ* P_{vanA}::P_{vanA}-*mipZ* P_{xylX}::P_{xylX}-*mipZ*$_{R242A}$-*eyfp*	This study
BH94	CB15N Δ*mipZ* P_{vanA}::P_{vanA}-*mipZ* P_{xylX}::P_{xylX}-*mipZ*$_{L248A}$-*eyfp*	This study
BH99	CB15N Δ*mipZ* P_{vanA}::P_{vanA}-*mipZ* P_{xylX}::P_{xylX}-*mipZ*$_{D42A}$-*eyfp*	This study
BH100	CB15N Δ*mipZ* P_{vanA}::P_{vanA}-*mipZ* P_{xylX}::P_{xylX}-*mipZ*$_{K13A}$-*eyfp*	This study
BH120	CB15N Δ*mipZ* P_{vanA}::P_{vanA}-*mipZ* P_{xylX}::P_{xylX}-*mipZ*$_{R196A}$-*eyfp*	This study
BH121	CB15N Δ*mipZ* P_{vanA}::P_{vanA}-*mipZ* P_{xylX}::P_{xylX}-*mipZ*$_{R221A}$-*eyfp*	This study
JAK10	CB15N Δ*mipZ* P_{vanA}::P_{vanA}-*mipZ* P_{xylX}::P_{xylX}-*mipZ*$_{T150A}$-*eyfp*	This study
LC40	MT190 P_{xylX}::P_{xylX}-*parA-eyfp*	This study
LC41	MT190 P_{xylX}::P_{xylX}-*parA*$_{R61A}$-*eyfp*	This study
LC42	MT190 P_{xylX}::P_{xylX}-*parA*$_{R194A}$-*eyfp*	This study
LC43	MT190 P_{xylX}::P_{xylX}-*parA*$_{R195A}$-*eyfp*	This study
LC44	MT190 P_{xylX}::P_{xylX}-*parA*$_{R221A}$-*eyfp*	This study
LC45	MT190 P_{xylX}::P_{xylX}-*parA*$_{V223A}$-*eyfp*	This study
LC46	MT190 P_{xylX}::P_{xylX}-*parA*$_{R224A}$-*eyfp*	This study
LC47	MT190 P_{xylX}::P_{xylX}-*parA*$_{K233A}$-*eyfp*	This study
LC48	MT190 P_{xylX}::P_{xylX}-*parA*$_{L236A}$-*eyfp*	This study
LC49	MT190 P_{xylX}::P_{xylX}-*parA*$_{L237A}$-*eyfp*	This study
LC50	MT190 P_{xylX}::P_{xylX}-*parA*$_{K241A}$-*eyfp*	This study
E. coli		
TOP10	F^- *mcr*A Δ(*mrr-hsd*RMS-*mcr*BC) Φ80*lac*ZΔM15 Δ*lac*X74 *rec*A1 *ara*D139 Δ(*ara leu*) 7697 *gal*U *gal*K *rps*L (Str^R) *end*A1 *nup*G	Invitrogen
Rosetta(DE3)pLysS	F^- *ompT hsdS*$_B$(r_B^- m_B^-) *gal dcm* (DE3) pLysSRARE (Cam^R)	Novagen

Table S2. Plasmids used in this work.

Plasmids	Description	Reference/Source
pBH57	pXYFPC-2 carrying *mipZ*$_{D147A}$	This study
pBH58	pXYFPC-2 carrying *mipZ*$_{E152A}$	This study
pBH8	pJET1.2 carrying *mipZ*	This study
pBH63	pXYFPC-2 carrying *mipZ*$_{R194A}$	This study
pBH64	pXYFPC-2 carrying *mipZ*$_{K197A}$	This study
pBH65	pXYFPC-2 carrying *mipZ*$_{R198A}$	This study
pBH68	pXYFPC-2 carrying *mipZ*$_{R219A}$	This study
pBH70	pXYFPC-2 carrying *mipZ*$_{L237A}$	This study
pBH71	pXYFPC-2 carrying *mipZ*$_{R242A}$	This study
pBH73	pXYFPC-2 carrying *mipZ*$_{L248A}$	This study
pBH78	pXYFPC-2 carrying *mipZ*$_{WT}$	This study
pBH79	pXYFPC-2 carrying *mipZ*$_{K13A}$	This study
pBH81	pXYFPC-2 carrying *mipZ*$_{D42A}$	This study
pBH94	pET21a(+) carrying *mipZ*$_{L237A}$	This study
pBH95	pET21a(+) carrying *mipZ*$_{R219A}$	This study
pBH97	pET21a(+) carrying *mipZ*$_{R194A}$	This study
pBH117	pET21a(+) carrying *mipZ*$_{R198A}$	This study
pBH119	pET21a(+) carrying *mipZ*$_{R242A}$	This study
pBH120	pET21a(+) carrying *mipZ*$_{L248A}$	This study
pBH130	pET21a(+) carrying *mipZ*$_{R221A}$	This study
pBH133	pET21a(+) carrying *mipZ*$_{K197A}$	This study
pBH137	pET21a(+) carrying *mipZ*$_{R196A}$	This study
pDK1	pET21a(+) carrying *parA*$_{WT}$	This study
pDK4	Plasmid carrying *mipZ*$_{K13A}$	(3)
pDK10	pET21a(+) carrying *mipZ*$_{K13A}$	(3)
pET21a(+)	Vector for overexpression of C-terminally His_6-tagged proteins, Amp^R	Novagen
pJAK10	pXYFPC-2 carrying *mipZ*$_{T150A}$	This study
pJET1.2	Plasmid for blunt-end ligation, Amp^R	Thermo Scientific
pLC62	pXYFPC-2 carrying *parA*$_{WT}$	This study
pLC63	pXYFPC-2 carrying *parA*$_{R61A}$	This study
pLC64	pXYFPC-2 carrying *parA*$_{R194A}$	This study
pLC65	pXYFPC-2 carrying *parA*$_{R195A}$	This study
pLC66	pXYFPC-2 carrying *parA*$_{R221A}$	This study
pLC67	pXYFPC-2 carrying *parA*$_{V223A}$	This study
pLC68	pXYFPC-2 carrying *parA*$_{R224A}$	This study
pLC69	pXYFPC-2 carrying *parA*$_{K233A}$	This study
pLC70	pXYFPC-2 carrying *parA*$_{L236A}$	This study
pLC71	pXYFPC-2 carrying *parA*$_{L237A}$	This study
pLC72	pXYFPC-2 carrying *parA*$_{K241A}$	This study
pMT182	pMR31 carrying *mipZ*, Cam^R	(10)
pMT244	pMT27 carrying *parA-eyfp*	This study
pMT362	Plasmid carrying *mipZ*$_{D42A}$	(10)
pMT415	pET21a(+) carrying *mipZ*$_{D42A}$	(10)
pTR26	pXYFPC-2 carrying *mipZ*$_{R196A}$	This study
pTR27	pXYFPC-2 carrying *mipZ*$_{R221A}$	This study
pXYFPC-2	Integrative plasmid for the expression of genes fused to *eyfp* at their 3' end under the control of P_{xyl}, Kan^R	(11)

Table S3. Oligonucleotides used in this work.

Oligonucleotide	Sequence [1]
CC2165-uni2	ttttCATATGgccgaaacgcgcgttatcgtcg
CC2165-rev2	ttGAGCTCctgcgccgccagcatcgtctcgcc
CC2165-rev7	tatGAGCTCcctgcgccgccagcatcgtctcgcc
CC2165-rev-HindIII	ccgcAAGCTTgtcgacggcctgcgccgccagcatcgtctcgcc
MipZ-rev	ttGAGCTCgcgccgccagcatcgtctcgccgga
ran14-lo	gcggcggcggcggc
ran14-up	gccgccgccgccgc
rand-Cy3	Cy3-gaggcagactagatcttctagttcgg
rand-biotin	Biotin-gaggcagactagatcttctagttcgg
rand-rev	ccgaactagaagatctagtctgcctc
GC56-biotin	Biotin-catacgaggtcgagatgc
GC56-rev	gcatctcgacctcgtatg
ATrich-biotin	Biotin-aataaataagaatagtaaaagtaaagaaag
ATrich-rev	ctttctttacttttactattcttatttatt
GCrich-biotin	Biotin-gcgccctgttcgccccgct
GCrich-rev	agcggggcgaacagggcgc
MipZD147A-for	ctgggcaccgttgctcccgtcaccctg
MipZD147-rev	cagggtgacgggagcaacggtgcccag
MipZT150A-for	cgttgatcccgtcgccctggagctgac
MipZT150A-rev	gtcagctccagggcgacgggatcaacg
MipZE152A-for	ccgtcaccctggcgctgaccaagcc
MipZE152A-rev	ggcttggtcagcgccagggtgacgg
MipZR194A-for	caccaccgaggcggcgaaccgcaagcgt
MipZR194A-rev	acgcttgcggttcgccgcctcggtggtg
MipZR196A-for	ccgaggcgcggaacgccaagcgtctggag
MipZR196A-rev	ctccagacgcttggcgttccgcgcctcgg
MipZK197A-for	ggcgcggaaccgcgcgcgtctggaggac
MipZK197A-rev	gtcctccagacgcgcgcggttccgcgcc
MipZR198A-for	gcggaaccgcaaggctctggaggaccgc
MipZR198A-rev	gcggtcctccagagccttgcggttccgc
MipZR219A-for	ggcccggcctggccgaccgcgtga
MipZR219A-rev	tcacgcggtcggccaggccgggcc
MipZR221A-for	ccggcctgcgcgacgccgtgatctatcgc
MipZR221A-rev	gcgatagatcacggcgtcgcgcaggccgg
MipZL237A-for	cagccacgcgcgggcgttctcgaagaac
MipZL237A-rev	gaaccgccgcgcggcgctggacaacaag
MipZR242A-for	cgccgcaggtggccccggtcccgg
MipZR242A-rev	ccgggaccggggccacctgcggcg
MipZL248A-for	ggtcccggtgtcggcgcagcatctggcg
MipZL248A-rev	cgccagatgctgcgccgacaccgggacc
ParA-uni	AAAACATAtgtccgctaatcctctccgcgttctgg
ParA-revHindIII	cggttcttgttctttgAAGCTTggcggccttggcc
ParAR61-for	ggccgcacccaggcccggaccacgctctatg
ParAR61-rev	gcgtggtccgggcctgggtgcggccaatg
ParAR194A-for	ctgaccatgtacgacgcccgcaacagcttgtc
ParAR194A-rev	gacaagctgttgcgggcgtcgtacatggtcagc
ParAR195-for	ccatgtacgaccgcgccaacagcttgtcgg
ParAR195-rev	ccgacaagctgttggcgcggtcgtacatgg
ParAR221-for	cgcggtgatcccggcgaacgtccgggtc
ParAR221-rev	gacccggacgttcgccgggatcaccgcg
ParAV223-for	cccgcggaacgcccgggtctccgaag
ParAV223-rev	cggagacccgggcgttccgcggg
ParAR224-for2	cccgcggaacgtcgcggtctccgaagcgccgtcg
ParAR224A-rev2	cgacggcgcttcggagaccgcgacgttccgcggg
ParAK233-for2	gccgtcgttcggcgcgcccgtgctgctctacg
ParAK233-rev2	gagcagcacgggcgcgccgaacgacggcgc
ParAL236-for	cggcaagcccgtggcgctctacgacctg
ParAL236-rev	caggtcgtagagcgccacgggcttgccg
ParAL237-for	ggcaagcccgtgctggcctacgacctgaaatg
ParAL237-rev	catttcaggtcgtaggccagcacgggcttgcc
ParAK241-for	ctgctctacgacctggcatgcgcgggtagcca
ParAK241-rev	tggctacccgcgcatgccaggtcgtagagcagcac

[1] Restriction sites are indicated in capital letters.

SUPPLEMENTAL DATASET

Dataset S1. **ChIP-seq analysis of the interaction of ParB and different MipZ variants with chromosomal DNA.** The file includes the statistics of the ChIP-seq analysis as well as the complete list of the binding sites obtained for the different proteins.

Dataset S2. **Raw data of the HDX-MS experiments.** The file includes the full list of peptides identified in the HDX-MS experiments for MipZ-D42A and ParA.

SUPPLEMENTAL EXPERIMENTAL PROCEDURES

Plasmid and strain construction

Plasmids containing mutant mipZ-eyfp alleles

The wild-type *mipZ* gene was PCR-amplified from pMT182 with primers CC2165-uni2 and CC2165-rev2. The reaction product was inserted into plasmid pJET1.2/blunt using the CloneJET PCR Cloning Kit (Thermo Scientific, USA). Subsequently, the ligation product (pBH8) was used as the template for site-directed mutagenesis, which was achieved by inverse PCR with suitable mutagenic primers. The mutant *mipZ* alleles were PCR-amplified from these resulting pBH8 derivatives using the forward primer CC2165uni-2 and the reverse primers MipZ-rev or CC2165-rev7. The products were cut with *Nde*I and *Sac*I and ligated into *Nde*I/*Sac*I-treated plasmid pXYFPC-2 (11). To generate plasmids encoding a wild-type MipZ-eYFP fusion or the corresponding K13A or D42A variants, plasmids pMT182 (10), pDK4 (3) and pMT362 (10) were digested with *Nde*I and *Sac*I and ligated into *Nde*I/*Sac*I-treated plasmid pXYFPC-2. The different plasmids (**Table S2**) were integrated at the chromosomal *xylX* locus of strain BH62 (Δ*mipZ* P_{van}::P_{van}-*mipZ*) by single-homologous recombination. The resulting strains (**Table S1**) were verified by colony PCR.

Plasmids for the overproduction of ParA-His$_6$, MipZ-His$_6$ and mutant MipZ variants in E. coli

The *parA* gene was PCR-amplified from *C. crescentus* genomic DNA using primers parA-uni and parA-revHindIII. The products were cut with with *Nde*I and *Hind*III and ligated into *Nde*I/*Hind*III-treated vector pET21a(+). The different *mipZ* alleles were PCR-amplified from the pXYFPC-2 derivatives described above using primers CC2165-uni-2 and CC2165-rev-HindIII. The products were cut with with *Nde*I and *Hind*III and ligated into *Nde*I/*Hind*III-treated vector pET21a(+).

Plasmids containing mutant parA-eyfp alleles

A fragment from plasmid pMT244 containing the wild-type *parA* gene fused in frame to *eyfp* was inserted between the *Nde*I and *Bsr*GI restriction sites of pXYFPC-2, yielding plasmid pLC62. Subsequently, pLC62 was used as a template for a site-directed mutagenesis, which was achieved by inverse PCR with appropriate mutagenic primers. The resulting plasmids were integrated at the chromosomal *xylX* locus of into *C. crescentus* strain MT190 (*parB*::*ecfp-parB*) by single-homologous recombination. The resulting strains (**Table S1**) were verified by colony PCR.

Growth conditions

C. crescentus CB15N and its derivatives were cultivated in PYE (peptone-yeast-extract) medium at 28 °C, supplemented with antibiotics when appropriate at the following concentrations (µg ml^{-1}; liquid/solid medium): kanamycin (5/25), streptomycin (5/5), spectinomycin (25/50). To induce the expression of genes placed under the control of the Pxyl or Pvan promoters, media were supplemented with 0.3% (w/v) D-xylose or 0.5 mM sodium vanillate, respectively. *E. coli* TOP10 (Invitrogen, USA) was used for cloning purposes. Proteins were overproduced in Rosetta (DE3) pLysS (Novagen, Germany). *E. coli* cells were cultivated aerobically in Luria-Bertani broth at 37 °C, supplemented when appropriate with 0.5% (w/v) glucose and antibiotics at the following concentrations (µg ml^{-1}; liquid/solid medium): ampicillin (200/200) or chloramphenicol (20/30). Protein overproduction was induced by addition of 1 mM isopropyl-β-D-thiogalactopyranoside (IPTG).

Light and fluorescence microscopy

Cells were immobilized on 1 % agarose pads and imaged using an Axio Imager.M1 microscope (Carl Zeiss AG, Germany) equipped with a Photometrics Cascade:1K EMCCD camera or a Zeiss Axio.Observer Z1 microscope equipped with a pco.edge 4.2 sCMOS camera (PCO). Images were acquired with a Zeiss Plan-Apochromat 100x/1.40 Oil Ph3 M27 objective. An X-Cite®120PC metal halide light source (EXFO, Canada) and an ET-YFP filter cube (Chroma, USA) were used for fluorescence imaging. Imaging data were analyzed with Metamorph 7.7 (Molecular Devices, USA) or Fiji 1.49 (12). Violin and boxplots were generated with R version 3.5.1 (http://www.r-project.org). The quantification of gradient patterns was performed in MATLAB R2014b (Mathworks, USA). To generate demographs, fluorescence intensity profiles were measured with Fiji and processed in R using the Cell Profiles script (13). Alternatively, automated demographic analyses were performed using BacStalk (14). Details of the FRAP analysis are provided in Supplemental Experimental Procedures.

Immunoblot analysis

Immunodetection was performed according to standard protocols using a polyclonal anti-MipZ antiserum (1:10,000) (10) or an affinity-purified polyclonal anti-GFP antibody (1:10,000) (#G1544; Sigma-Aldrich, Germany). Immunocomplexes were detected with a secondary goat anti-rabbit

antibody conjugated with horseradish peroxidase and visualized with the Western Lightning plus - ECL chemiluminescent reagent (PerkinElmer, USA) in a ChemiDoc MP imaging system (Bio-Rad Laboratories, USA). Images were aquired in ImageLab 5.0 (Bio-Rad Laboratories, USA) and processed in Adobe Illustrator CS5 (Adobe Systems, USA).

Microscale thermophoresis

Proteins were serially diluted 16 times with EMSA buffer containing 0.46 mM ATPγS and mixed with a fluorescently (Cy3-) labeled 26 bp-long dsDNA oligonucleotide (rand-cy3 and rand-rev; Eurofins MWG Operon, Germany). The solutions were then transferred into Hydrophilic Capillaries (NanoTemper, Germany) and analyzed in a Monolith NT.115 device (NanoTemper, Germany). The measurements were performed with 20-40 % LED power and 20 % MST power. The concentration of the labeled dsDNA oligonucleotide was adjusted such that the fluorescence signal was in the range of 300-1,000 units.

FRAP analysis

Fluorescence-recovery-after-photobleaching (FRAP) analysis was performed using a Zeiss Axio.Observer Z1 microscope equipped with a 488 nm-solid state laser and a 2D-VisiFRAP Galvo System multi-point FRAP module (Visitron Systems, Germany), using 300-ms pulses at a laser power of 15%. After acquisition of a pre-bleach image, cells were imaged every 1.75 s (BH85, BH86, BH100) or every 4 s (BH64). For each time point, the integrated fluorescence intensities of the whole cell, the bleached region and an equally sized unbleached region were measured using Fiji 1.49 (12). Recovery half-times were calculated as described previously (3) by fitting the data to a single-exponential function in QtiPlot 0.9.9 (www.qtiplot.com).

ChIP-seq analysis

To compare the DNA-binding profiles of MipZ and ParB, *C. crescentus* wild-type strain NA1000 was grown to an OD_{660} of 0.5 in PYE medium (80 ml per sample). The medium was then supplemented with 10 µM sodium phosphate buffer (pH 7.6) and treated with formaldehyde (1% final concentration) for 10 min at room temperature to achieve crosslinking. Subsequently, the cultures were incubated for an additional 30 min on ice and washed three times in phosphate-buffered saline (PBS, pH 7.4). The resulting cell pellets were stored at -80°C. After resuspension in TES buffer (10 mM Tris-HCl pH 7.5, 1 mM EDTA, 100 mM NaCl) containing 10 mM dithiothreitol (DTT), the cells were incubated for 10 min at 37°C in the presence of Ready-Lyse lysozyme solution (Epicentre, Madison, WI, USA) according to the manufacturer's instructions. The lysates were sonicated (Bioruptor® Pico) at 4°C using 15 bursts of 30 sec to shear DNA fragments to an average length of 0.3-0.5 kb and cleared by centrifugation at 14,000 rpm for 2 min at 4°C. The volume of the lysates was then adjusted (relative to the protein concentration) to 1 ml with ChIP buffer (16.7 mM Tris-HCl pH 8.1, 0.01% SDS, 1.1% Triton X-100, 1.2 mM EDTA, 167 mM NaCl) containing protease inhibitors (Roche, Switzerland) and pre-cleared with 80 µl of Protein-A agarose (Roche) and 100 µg BSA. The pre-cleared lysates were then incubated overnight at 4°C with polyclonal rabbit antibodies targeting ParB (1:500 dilution) or MipZ (1:500 dilution) (10). The immunocomplexes were captured by incubation with Protein-A agarose beads (pre-saturated with BSA) for 2 h at 4°C. The beads were washed with low-salt washing buffer (20 mM Tris-HCl pH 8.1, 0.1% SDS, 1% Triton X-100, 2 mM EDTA, 150 mM NaCl), with high-salt washing buffer (20 mM Tris-HCl pH 8.1, 0.1% SDS, 1% Triton X-100, 2 mM EDTA, 500 mM NaCl), with LiCl washing buffer (10 mM Tris-HCl pH 8.1, 0.25 M LiCl, 1% NP-40, 1% deoxycholate, 1 mM EDTA) and finally twice with TE buffer (10 mM Tris-HCl pH 8.1, 1 mM EDTA). Subsequently, immunocomplexes were eluted from the Protein-A agarose beads with two times 250 µL elution buffer (1% SDS, 0.1 M $NaHCO_3$, freshly prepared) and incubated overnight with 300 mM NaCl at 65°C to reverse the crosslinks. The samples were then treated with 2 µg of Proteinase K for 2 h at 45°C in 40 mM EDTA and 40 mM Tris-HCl (pH 6.5). DNA was extracted using phenol:chloroform:isoamyl alcohol (25:24:1), ethanol-precipitated using 20 µg of glycogen as a carrier and resuspended in 50 µl of DNAse/RNAse-free water.

To determine the chromosomal DNA-binding profiles of wild-type, monomeric and dimeric MipZ, overnight cultures of *C. crescentus* NA1000 (negative control sample), BH64 ($MipZ_{WT}$-eYFP), BH99 ($MipZ_{D42A}$-eYFP) and BH100 ($MipZ_{K13A}$-eYFP) were grown in PYE medium supplemented with vanillate (500 µM). The cells were harvested by centrifugation, washed three times with PYE medium, and then cultivated in 80 ml of PYE medium (starting OD_{660} ~ 0.05) for 5 h at 30°C to deplete the native MipZ protein. Subsequently, 0.3% xylose was added to induce the expression of the different *mipZ-eyfp* fusions from the P_{xyl} promoter, and the cultures were incubated for an additional 1 h at 30 °C. Finally, the cultures (final OD_{660} ~ 0.6) were treated with formaldehyde (1% final concentration) in 10 µM sodium phosphate buffer (pH 7.6) for 10 min at room temperature to achieve cross-linking. The cells were incubated for an additional 30 min on ice, washed three times in phosphate-buffered saline (PBS, pH 7.4) and stored at -80°C. After resuspension in Dilution buffer (GFP-Trap®_A for Immunoprecipitation of GFP-Fusion Proteins, ChromoTek, Germany; 10 mM Tris-HCl pH 7.5, 0.5 mM EDTA, 150 mM NaCl), the cells were incubated in the presence of Ready-Lyse lysozyme solution (Epicentre, Madison, WI) for 10 minutes at 37°C according to the manufacturer's instructions. The lysates were sonicated (Bioruptor® Pico) at 4 °C using 15 bursts of 30 sec to shear DNA fragments to an average length of 0.3-0.5 kbp and cleared by centrifugation at 14,000 rpm for 2 min at 4 °C. The volume of the lysates was then adjusted (relative to the protein concentration) to 1 ml using Dilution buffer supplemented with protease inhibitors (Roche). Cleared lysates were incubated for 2 h at 4 °C with GFP-Trap®_A agarose beads (pre-washed three times with Dilution buffer). Beads were then washed consecutively with Dilution buffer, twice with high-salt Wash buffer (10 mM Tris-HCl pH 7.5, 0.5 mM EDTA, 500 mM NaCl) and finally twice with TE buffer (10 mM Tris-HCl pH 8.1, 1 mM EDTA). The captured immuno-complexes were eluted from the GFP-Trap®_A agarose beads with two times 250 µL of Elution buffer (1% SDS, 0.1 M $NaHCO_3$, freshly prepared) and

incubated overnight with 300 mM NaCl at 65 °C to reverse the crosslinks. The samples were then treated with 2 µg of Proteinase K for 2 h at 45 °C in 40 mM EDTA and 40 mM Tris-HCl (pH 6.5). DNA was extracted using phenol:chloroform:isoamyl alcohol (25:24:1), ethanol-precipitated using 20 µg of glycogen as a carrier and resuspended in 50 µl of DNAse/ RNAse-free water.

Immunoprecipitated chromatin was used to prepare sample libraries used for deep-sequencing at Fasteris SA (Geneva, Switzerland). ParB/MipZ and MipZ-eYFP ChIP-Seq libraries were prepared using the Chrysalis 36cycles v 4.0 Sequencing Kit and the DNA Sample Prep Kit (Illumina, Switzerland), respectively, according to the manufacturer's instructions. Single-end runs were performed on an Illumina Genome Analyzer IIx (38 cycles: ParB/MipZ) or an Illumina HiSeq2500 (50 cycles: MipZ-eYFP fusions and NA1000 negative control) instruments, yielding several million reads. The single-end sequence reads (stored as fastq files) were mapped (Map_with_Bowtie_for_ Illumina_V1.1.2, -m 1 modified parameter) to the *C. crescentus* NA1000 genome sequence (NC_011916.1) using the web-based analysis platform Galaxy (https://usegalaxy.org). ChIP-Seq read sequencing and alignment statistics are summarized in **File S1**. The standard genomic position format files (BAM, using Samtools, http://samtools.sourceforge.net) were imported into SeqMonk version 1.45.4 (http://www. bioinformatics. babraham.ac.uk/projects/seqmonk) to build ChIP-Seq normalized sequence read profiles. Briefly, the genome was subdivided into 50 bp probes, and for every probe, we calculated the number of reads per probe as a function of the total number of Reads Per Million (RPM, using the Read Count Quantitation option). MipZ-eYFP ChIP-Seq traces are normalized (ratio) *versus* the NA1000 (WT) negative control sample. The processed data, shown in part in **Fig. 3 and S6**, are provided in **File S1**. Sequence data have been deposited to the Gene Expression Omnibus (GEO) database (accession number GSE137346, samples numbers GSM4076063–GSM4076066 and GSM4086387). The raw data of the ParB ChIP-Seq analysis have been previously deposited to the GEO database (accession number GSE79918, sample GSM2108314) (15).

SUPPLEMENTAL REFERENCES

1. Pei, J. and Grishin, N.V. (2014) PROMALS3D: multiple protein sequence alignment enhanced with evolutionary and three-dimensional structural information. *Methods Mol. Biol.*, **1079**, 263-271.
2. Waterhouse, A.M., Procter, J.B., Martin, D.M., Clamp, M. and Barton, G.J. (2009) Jalview Version 2 – a multiple sequence alignment editor and analysis workbench. *Bioinformatics*, **25**, 1189-1191.
3. Kiekebusch, D., Michie, K.A., Essen, L.O., Löwe, J. and Thanbichler, M. (2012) Localized dimerization and nucleoid binding drive gradient formation by the bacterial cell division inhibitor MipZ. *Mol. Cell*, **46**, 245-259.
4. Chu, C.H., Yen, C.Y., Chen, B.W., Lin, M.G., Wang, L.H., Tang, K.Z., Hsiao, C.D. and Sun, Y.J. (2019) Crystal structures of HpSoj-DNA complexes and the nucleoid-adaptor complex formation in chromosome segregation. *Nucleic Acids Res.*, **47**, 2113-2129.
5. Schumacher, M.A., Henderson, M. and Zhang, H. (2019) Structures of maintenance of carboxysome distribution Walker-box McdA and McdB adaptor homologs. *Nucleic Acids Res.*, **47**, 5950-5962.
6. Wu, W., Park, K.T., Holyoak, T. and Lutkenhaus, J. (2011) Determination of the structure of the MinD-ATP complex reveals the orientation of MinD on the membrane and the relative location of the binding sites for MinE and MinC. *Mol. Microbiol.*, **79**, 1515-1528.
7. Kelley, L.A., Mezulis, S., Yates, C.M., Wass, M.N. and Sternberg, M.J. (2015) The Phyre2 web portal for protein modeling, prediction and analysis. *Nat. Protoc.*, **10**, 845-858.
8. Zhang, H. and Schumacher, M.A. (2017) Structures of partition protein ParA with nonspecific DNA and ParB effector reveal molecular insights into principles governing Walker-box DNA segregation. *Genes Dev.*, **31**, 481-492.
9. Evinger, M. and Agabian, N. (1977) Envelope-associated nucleoid from *Caulobacter crescentus* stalked and swarmer cells. *J. Bacteriol.*, **132**, 294-301.
10. Thanbichler, M. and Shapiro, L. (2006) MipZ, a spatial regulator coordinating chromosome segregation with cell division in *Caulobacter*. *Cell*, **126**, 147-162.
11. Thanbichler, M., Iniesta, A.A. and Shapiro, L. (2007) A comprehensive set of plasmids for vanillate- and xylose-inducible gene expression in *Caulobacter crescentus*. *Nucleic Acids Res.*, **35**, e137.
12. Schindelin, J., Arganda-Carreras, I., Frise, E., Kaynig, V., Longair, M., Pietzsch, T., Preibisch, S., Rueden, C., Saalfeld, S., Schmid, B. *et al.* (2012) Fiji: an open-source platform for biological-image analysis. *Nat. Methods*, **9**, 676-682.
13. Cameron, T.A., Anderson-Furgeson, J., Zupan, J.R., Zik, J.J. and Zambryski, P.C. (2014) Peptidoglycan synthesis machinery in *Agrobacterium tumefaciens* during unipolar growth and cell division. *MBio*, **5**, e01219-01214.
14. Hartmann, R., van Teeseling, M.C.F., Thanbichler, M. and Drescher, K. (2018) BacStalk: a comprehensive and interactive image analysis software tool for bacterial cell biology. *bioRxiv*, doi: 10.1101/360230.
15. Berge, M., Campagne, S., Mignolet, J., Holden, S., Theraulaz, L., Manley, S., Allain, F.H. and Viollier, P.H. (2016) Modularity and determinants of a (bi-)polarization control system from free-living and obligate intracellular bacteria. *Elife*, **5**, e20640.
